# Complete genome sequence of *Pseudomonas stutzeri* S116 owning bifunctional catalysis provides insights into affecting performance of microbial fuel cells

**DOI:** 10.1186/s12866-022-02552-8

**Published:** 2022-05-19

**Authors:** Peng Li, Wenfeng Yuan, Yitie Huang, Caiyu Zhang, Chide Ni, Qi Lin, Zhihuang Zhu, Jianxin Wang

**Affiliations:** 1 School of Ocean Science and Technology, Zhejiang Ocean University, No.1, Haida South Road, Lincheng Changzhi Island, Zhoushan, Zhejiang 316022 People’s Republic of China; 2 Fisheries Research Institute of Fujian Province, Xiamen, 361013 China

**Keywords:** Biocatalyst, Microbial fuel cells, *Pseudomonas stutzeri*, Complete genome

## Abstract

**Background:**

*Pseudomonas stutzeri* S116 is a sulfur-oxidizing bacteria isolated from marine sludge. It exhibited excellent electricity generation as bioanode and biocathode applied in microbial fuel cells (MFCs). Complete genome sequencing of *P. stutzeri* and cyclic voltammetry method were performed to reveal its mechanism in microbial fuel cells system.

**Results:**

This study indicated that the MFCs generated a maximum output voltage of 254.2 mV and 226.0 mV, and maximum power density of 765 mW/m^2^ and 656.6 mW/m^2^ respectively. Complete genome sequencing of *P. stutzeri* S116 was performed to indicate that most function genes showed high similarities with *P. stutzeri*, and its primary annotations were associated with energy production and conversion (6.84%), amino acid transport and metabolism (6.82%) and inorganic ion transport and metabolism (6.77%). Homology of 36 genes involved in oxidative phosphorylation was detected, which suggests the strain S116 possesses an integrated electron transport chain. Additionally, many genes encoding pilus-assembly proteins and redox mediators (riboflavin and phenazine) were detected in the databases. Thiosulfate oxidization and dissimilatory nitrate reduction were annotated in the sulfur metabolism pathway and nitrogen metabolism pathway, respectively. Gene function analysis and cyclic voltammetry indicated that *P. stutzeri* probably possesses cellular machinery such as cytochrome *c* and redox mediators and can perform extracellular electron transfer and produce electricity in MFCs.

**Conclusion:**

The redox mediators secreted by *P. stutzeri* S116 were probably responsible for performance of MFCs. The critical genes and metabolic pathways involved in thiosulfate oxide and nitrate reduction were detected, which indicated that the strain can treat wastewater containing sulfide and nitrite efficiently.

**Supplementary Information:**

The online version contains supplementary material available at 10.1186/s12866-022-02552-8.

## Background

Sulfur-oxidizing bacteria (SOB) can oxidize sulfur compounds as energy sources and utilize inorganic carbon (CO_2_) for their growth [1]. Therefore, they play an important role in environmental remediation, removing pollutants containing reduced sulfide and fixing CO_2_ [2]. Previously reported SOB belongs to the genera *Thiomonas*, *Acidithiobacillus*, *Thiothrix*, *Pseudomonas*, *Thiobacillus*, *Halothiobacillus*, *Chlorobium*, *Rhodospirillum* and *Sulfurimonas* [3–5]. Currently, studies of SOB have mainly focused on metal recovery from minerals [6], removal of high concentrations of H_2_S from biogas and industrial waste gas [7, 8], and wastewater treatment containing reduced sulfur compounds (S^2−^, S_2_O_3_^2−^, S) [9]. However, few studies have reported the application of SOB in microbial fuel cells (MFCs) and the related mechanisms have rarely been investigated.

Microbial fuel cell (MFC) is a promising technology for treating wastewater and pollutants and directly convert chemical energy into electrical energy [10, 11]. The large-scale application of MFCs is limited by electrode materials and electrogenic microorganisms [12]. Compared with costly materials, microorganisms with excellent electrocatalytic activity are more practical and inexpensive in enhancing MFCs performance.

The genus *Pseudomonas* is a common sulfur-oxidizing bacterium that possesses strong adaptability to environments with low dissolved oxygen and high concentrations of organic substances and shows excellent performance for odorous river bioremediation [13]. Utilizing oxygen as the electron acceptor, *Pseudomonas* can remove high concentrations of H_2_S effectively in biogas by biotricking filters [7]. In addition, *Pseudomonas* is a typical electrochemically active bacterium used in MFCs [14], and it was found to be important for 2,4-DCP degradation and could affect bioelectrochemical activities and MFCs performance. Luo et al. introduced an exogenous global regulator *IrrE* into *Pseudomonas aeruginosa*, which enhanced the power density of air-cathode MFCs [15].

It is generally believed that the mechanism of electricity generation in two dual microbial fuel cells is as follows: Electroactive microorganisms in the anodic chamber oxidize substrates (pollutants or organic substances) to generate electrons, and then the electrons are carried by an external electric circuit to a cathode (reduction reaction). Therefore, a current flows and produces electrical energy [16, 17]. Clearly, extracellular electron transfer (EET) efficiency is an essential element between electricity-producing microorganisms and an anode affecting MFCs performance. Current studies provide three basic mechanisms of EET: 1. c-type cytochromes. Electroactive microorganisms possessing cytochromes can transfer electrons from the cell metabolism to outside of the cell [18, 19]. 2. Nanowires. Special bacterial pili play an important role in EET [20, 21]. 3. Redox mediators. Riboflavin can promote electron transfer from electroactive bacteria to anodes in MFCs [22]. As a significant member of anodic electrogenic bacteria, *Pseudomonas* can directly or indirectly transfer electrons to an anode surface (via cytochrome c/bacterial pili or its external mediator) [23–25]. The external mediator can facilitate EET toward the anode, improve the performance of the anode and promote anaerobic survival of *Pseudomonas* in MFCs (most *Pseudomonas* are facultative anaerobic and aerobic bacteria) [26, 27].

*Pseudomonas* successfully used in MFCs is attributed to its great biodegradation capabilities [28–32], and it is considered to be an excellent anode biocatalyst [33, 34]. However, the application of *Pseudomonas* as a bio-cathode in MFCs has rarely been reported. The researchs of bio-cathode are mainly focused on the microorganisms of mixed culture rather than pure culture strains. It is difficult to interpret the mechanism underlying electricity generation at a bio-cathode electrode. Difference from anode electrogenic microorganisms, the cathode microorganisms are used as bio-catalysts to accept electrons from the cathode electrode [35], and the biofilm on cathode surface efficiently enhances electron density [36]. Consequently, bio-cathode MFCs have attracted much attention as the promising technology applied in energy recovery. At present, bio-cathodes are classified as follows: (1) Oxygen. Due to the low-cost and high redox potential, oxygen is considered as an excellent terminal electron acceptor. Cathodic bacteria on the electrode can catalyze the reduction of oxygen to generate electricity [37]. (2) Inorganic salts. Nitrate and sulfate directly accept electrons through microbial metabolism (denitrifying bacteria and sulfate reducting bacteria) [38]. (3) Others. fumarate, urea, carbon dioxide and redox mediators can be used as electron acceptor [39]. In brief, biological cathode MFCs can use electrogenic microorganisms as catalysts to prompt the electron transfer.

This study investigates the function of bioelectricity generation of *P. stutzeri* S116 as an anodic and cathodic biocatalyst affecting the performance of MFCs. First, a *P. stutzeri* S116 strain was isolated from the marine sludge. Second, the electrochemical activity of the strain in MFCs was investigated. Third, complete genome of the strain S116 was sequenced and its functions were annotated and analyzed. The genetic functions of *P. stutzeri* related to electron transfer were illustrated, which may interpret the mechanism of microbial catalysis in MFCs.

## Methods

### Sample preparation and strain screening

Marine activated sludge samples were collected from an anaerobic pool with the constant temperature of 35 °C in a marine sewage treatment plant. Fifty milliliters of sludge samples were suspended in 450 mL of sterile seawater and mixed with a dilution ratio of 10^− 1^. The suspension was collected and stored at 4 °C for enrichment and isolation. The enrichment culture medium for SOB (ECMS) in 1 L of sterile seawater contained the following components: 10 g of Na_2_S_2_O_3_·5H_2_O, 4.0 g of KH_2_PO_4_, 4.0 g of K_2_HPO_4_, 0.8 g of MgSO4·7H_2_O, 0.4 g of NH_4_Cl, and 10 mL of trace elements. One liter of the trace element solution included the following: 50.0 g of EDTA, 22.0 g of ZnSO_4_·7H_2_O, 5.54 g of CaCl_2_, 5.06 g of MnCl_2_·4H_2_O, 4.99 g of FeSO_4_·7H_2_O, 1.10 g of (NH_4_)_2_MoO_4_·4H_2_O, 1.57 g of CuSO_4_·5H_2_O, 1.61 g of CoCl_2_·6H_2_O, 1 L of sterile seawater. Agar (1.5–2%) added as a solidifying agent was used to screen SOB.

A 5 mL aliquot of the prepared sample was inoculated into flasks containing 45 mL of ECMS medium and cultivated at 35 °C with a rotation speed of 120 r/min for 3 days. After three successive cultures, 0.1 mL of the enrichment samples were spread onto agar ECMS plates and incubated at 35 °C for 3 days. Subsequently, colonies were picked and streaked onto fresh agar ECMS plates three times, and purified isolates were obtained and inspected by an optical microscope. General features of *P. stutzeri* S116 is shown in Table 1. The purified strains were activated in LB fluid medium for 12–24 h (OD_600_ = 0.6–0.8), then 1 mL bacterial suspension was inoculated into 80 mL anolyte. To screen excellcent electrochemically active bacteria (EAB), the output voltage higher than 200 mV is suitable for MFCs.

**Table 1:** General features of *Pseudomonas*

Items	Description
**General features**	
Classification	Domain *Bacteria*
	Phylum *Proteobacteria*
	Class *Gammaproteobacteria*
	Order *Pseudomonadaceae*
	Genus *Pseudomonas*
Gram stain	Negative
Cell shape	Rod
Motility	Motile
Pigmentation	No-pigmented
Investigation type	Bacteria
Project name	*Pseudomonas stutzeri* S116
**Sampling reference**	
Sampling location	Zhoushan, Zhejiang, China 29.916303 N 122.390636 E
Source	Activated sludge
**Isolation conditions**	
Source of carbon	EDTA
Salt concentration	3% (w/v) NaCl
Temperature of incubation	35 °C
pH	7.0
**Sequencing**	
Sequencing method	Illumina HiSeq
Assembly	De Novo *Assembly*

### Morphological characteristic

EBA adsorbed on the electrodes was collected and immobilized in 2.5% glutaraldehyde solution, then deposited in 4 °C refrigerator for 24 h. The prepared sample was submitted to Beijing Zhong Ke Bai Ce Technology Co., LTD. The morphological characteristic of S116 was analysed by scanning electron microscopy (SEM) using secondary electron image technology. Before scanning, metal spraying was operated to immobilize the sample.

### MFC configuration

A double chamber MFC was configured with cylindrical glass. The chambers were separated by a cation exchange membrane Nafion117 (5 cm × 5 cm, DuPont, USA). Each chamber had a volume of 100 mL. Reactor 1: The anodes were assembled from a carbon cloth (1.5 cm × 1 cm Hesen HCP330N, Shanghai, China), the strain S116 used as the anode catalyst was inoculated in the anodic medium with a volume of 80 mL, the anodic medium in 1 L of artificial seawater contained the following: 0.0352 g of KH_2_PO_4_, 0.128 g of NaCl, 0.01 g of FeSO_4_·7H_2_O, 0.188 g of (NH_4_)_2_SO_4_, 0.2 g of NaHCO_3_, 0.18 g of MgSO4·7H_2_O, 0.05 g of CaCl_2_, 0.73 g of KNO_3_, and 5 g of NaS_2_O_3_. Potassium ferricyanide (50 mM, 80 mL) was used as the catholyte, and a carbon cloth was used as the cathode electrode. Reactor 2: The anode electrodes assembled carbon cloth were equipped in the MFC. Anaerobic activated sludge from a marine sewage treatment plant (Zhoushan, China) was used as the anodic inoculum. Before running the reactions, the anode chamber was filled with 40 mL of sludge and 40 mL of a mixture of substrate and medium. The anodic medium of 1 L artificial seawater contained 0.8787 g of CH_3_COONa, 0.361 g of KNO_3_, 0.0255 g of KH_2_PO_4_, 0.0427 g of K_2_HPO_4_·3H_2_O, and 1 mL of trace elements at pH 7.0, the cathodic medium is as same as the anodic medium in Reactor 1. The anode and cathode electrodes were connected by an external copper wire with a 900 Ω resistance. The MFC was operated at 25 °C. All experimental reactions were performed in triplicate to ensure reproducibility.

### MFC performance analysis

The output voltage of the MFC was recorded by a data acquisition system. Polarization curves and power density curves were calculated by Ohm’s law, which was obtained by changing external resistors. Ohm’s law was described as follows: I (A/m^2^) = U/(RA) and P (W/m^2^) = U^2^/(RA), where I is the current density, R is the resistance, P is the power density, U is the voltage, and A is the area of the cathode.

Cyclic voltammetry (CV) measurements of electrodes were operated by the three-electrode system using an electrochemical workstation (Bio-Logic, SP-300, France). The carbon cloth, platinum electrode and saturated calomel electrode were used as the working, reference and counter electrodes, respectively. CV was performed at a scanning speed of 50 mV/s from − 1 to 1.0 V in Reactor 1 (− 1 to 0.2 V in Reactor 2). EIS was carried out at a sinusoidal perturbation amplitude of 5 mV in a frequency range from 100 kHz to 5 mHz.

### Identification of bacterial species

The screened electrochemically active bacteria (EAB) S116 was identified using 16S rRNA gene sequencing. The DNA was extracted by a bacterial genome DNA extraction kit (Ezup, Sangon Biotech, Shanghai), and the 16S rRNA gene was amplified by PCR (2720 thermal cycler, Applied Biosystems) with universal primers (7F: 5′-CAGAGTTTGATCCTGGCT-3′, 1540R: 5′-AGGAGGTGATCCAGCCGCA -3′) [40]. The loop condition of PCR was as follows: predenaturation for 4 min at 94 °C, 30 cycles of denaturation at 94 °C for 45 s, annealing at 55 °C for 45 s, elongation at 72 °C for 60 s, repair extension at 72 °C for 8 min, and termination reaction at 4 °C. PCR products were purified using 1% agarose gel electrophoresis and subjected to Sanger sequencing (Sangon Biotech (Shanghai) Co., Ltd.). The sequencing results were aligned using BLAST, and phylogenetic trees were constructed by MEGA (MEGA version 7.0) to analyze 16S rRNA gene sequences [41].

### Complete genome sequence and functional annotation of *P. stutzeri*

High-quality genomic DNA of *P. stutzeri* was extracted using a QIAGEN Genomic tip (Biomarker Technologies Co., Ltd.). The concentration and purity of DNA were detected using a NanoDrop and Qubit (Thermo Scientific, USA), and large segments were filtered using the BluePippin system (Sage Science, USA). A library was prepared using the large segments DNA, Oxford Nanopore Technologies (ONT) Template prep kit (SQK-LSK109) and NEB Next FFPE DNA Repair Mix kit. The high-quality library was sequenced on the ONT PromethION platform, and the raw sequencing data were obtained.

For genome assembly, quality control of the sequencing data was performed by Guppy3.2.6 software to filter low-quality fragments of the reads. The obtained subreads were assembled using Canu v1.5/ wtdbg v2.2 [42]. For genome component prediction, coding DNA sequences (CDSs) were predicted using Prodiga V2.50 [43]. tRNAs, rRNAs and ncRNAs were predicted using tRNAscan-SE v1.3.1 [44] and Infernal v1.1 (based on RFAM v12.0 database), respectively. For functional annotation, the predicted gene sequences from Prodiga were aligned by BLAST v2.2.29 [45] against the functional databases of Cluster of Orthologous Groups (COG) [46], Kyoto Encyclopedia of Genes and Genomes (KEGGs) [47], Swiss-Prot [48], Non-Redundant Protein Database (Nr), Gene Ontology (GO) [49], Carbohydrate-active enzymes database (CAZy) [50], transporter classification database (TCDB) [51], and virulence factor database (VFDB) [52]. The Nr database contains comprehensive protein sequences and annotation information. GO unifies the gene products of all species in different databases. KEGG annotation of metabolic pathways in *Pseudomonas stutzeri* S116. Nonredundant protein sequences with high quality are manually annotated using the Swiss-Prot database, and the annotation results have corresponding experimental verification with high reliability. Five types of functional carbohydrate-active enzymes are collected in CAZy database, TCDB contains protein sequences of various transporters, while the virulence factors in pathogenic bacteria are annotated in VFDB.

## Results

### Morphological characteristic of *P. stutzeri*

**Fig. 1:**
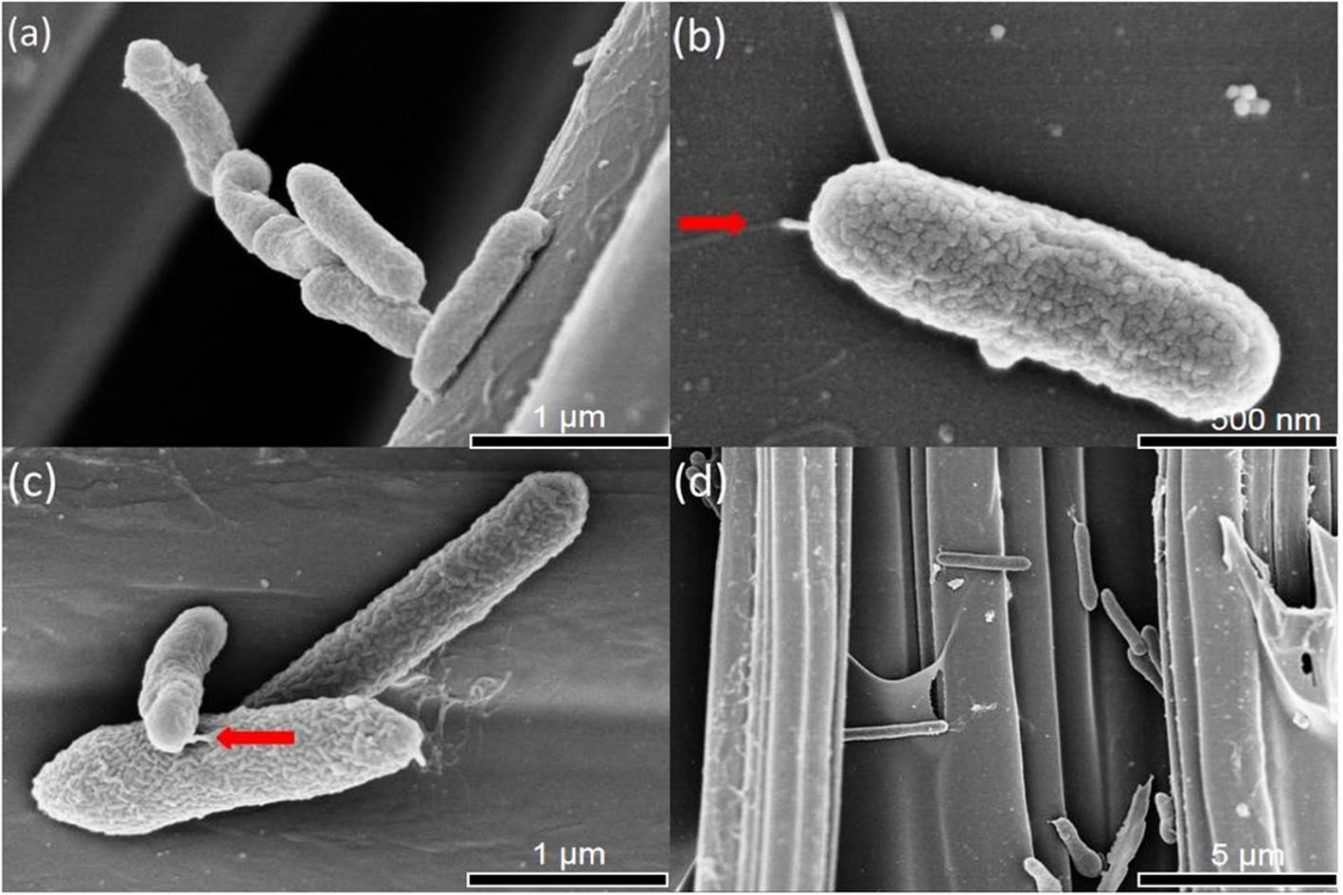
The surface morphology in electrodes was observed by SEM in different resolution. The magnification of **a** and **c** was 50,000, **b** was 70,000, **d** was 5000. Red arrow: flagella

The morphology of the isolated strain was characterized by (SEM). SEM shows that the strain adhered to the surface of the electrode. Strain S116 was a short rod without spores, the length and diameter of the bacterial cell were approximately 1.5 μm and 0.5 μm, respectively, its surface was wrinkled, and pili were observed. The biofilm was surveyed among carbon fibers. SEM data associated with this article is shown in Fig. 1.

### Phylogenetic analysis

Generally, it is difficult to differentiate closely related species based on 16S rRNA gene sequencing. Besides 16S rRNA gene, *gyrB*, *rpoB*, and *rpoD* have been used as housekeeping genes in taxonomic studies of genus *Pseudomonas* [53]*.* Simultaneously, complete genome sequence of strain S116 provides the three valuable gene sequences, which contributes to enhancing accuracy on identification of bacteria.

**Fig. 2:**
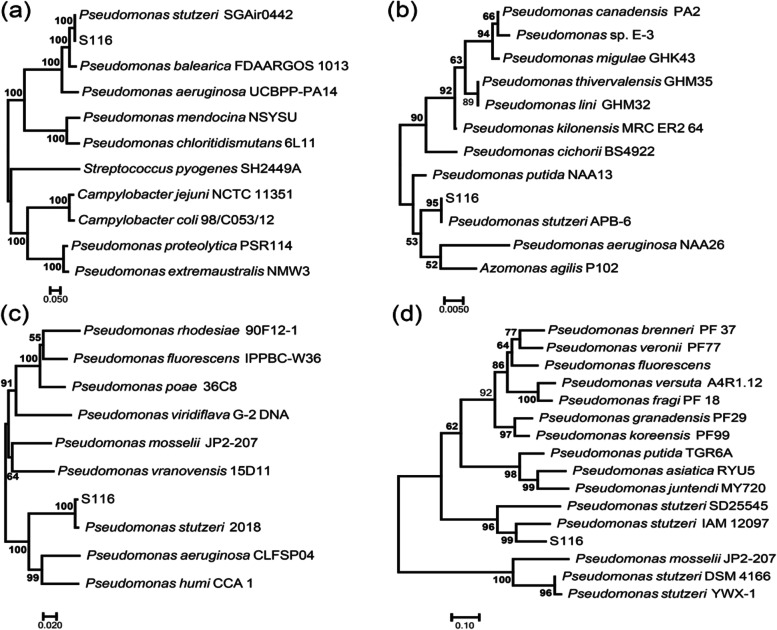
Phylogenetic tree based on **a** *rpoB*, **b** 16S rRNA, **c** *gyrB* and **d** *rpoD.* The phylogenetic tree was constructed using MEGA 7.0 with the neighbor-joining method. a phylogenetic test was performed using the bootstrap method (1000 replicates), and the evolutionary distance among similar sequences was computed using the Kimura 2-parameter model. Values above 50% on the condensed tree are shown at each node

Sequences alignment were performed using BLAST. Aligned sequences with similarities were selected to construct phylogenetic trees using MEGA 7.0. The sequence data of *P. stutzeri* S116 are publicly available in the NCBI database (GenBank accession number MZ220459, BioProject accession PRJNA743140). The phylogenetic trees based on the four housekeeping genes were exhibited in Fig. 2. The result indicated the strain S116 has closely homology with genus *Pseudomonas stutzeri.*

### Electrochemical property of the bioanode and biocathode

**Fig. 3:**
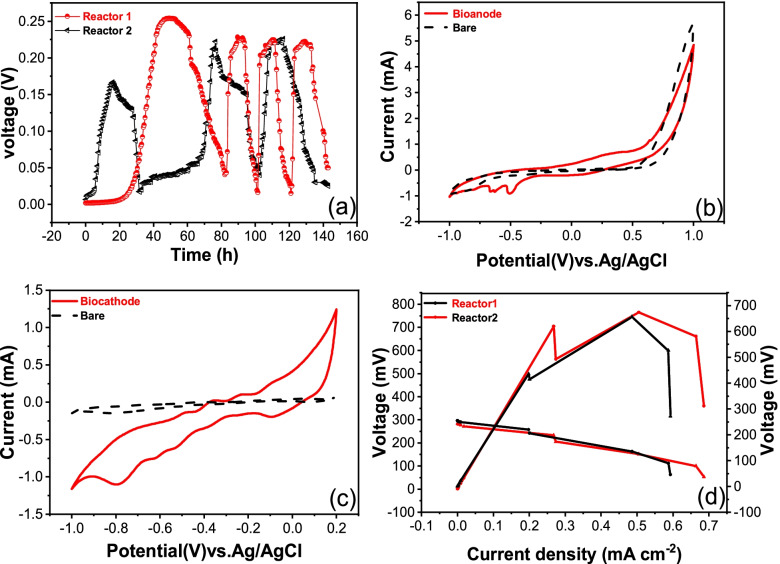
Voltage output (**a**), Cyclic voltammogram (CV) of the bioanode (**b**) and the biocathode (**c**), power density and polarization curves of MFC (**d**)

Thirty milliliters of the anodic medium was replaced by fresh one when the output voltage of the MFC was reduced to approximately 50 mV. After the MFC was operated for 30 h, the Reactor 2 reached the stable generation voltage in the first cycle with peak voltage at 170.5 mV, and for 80 h the Reactor 1 reached the highest peak voltage at 254.2 mV. During the second and third cycles, the Reactor 1 and 2 reached the highest output voltages of 228.3 mV and 225.5 mV, respectively. It took less than 10 hours for the Reactor 1 to produce an output voltage from the lowest voltage to the highest voltage (Fig. 3a).

To investigate the mechanisms of electrogenic microorganism in MFCs, CV analysis of bioanode and biocathode were performed (shown in Fig. 3 b and c). The bioelectrode compared with the bare carbon cloth electrode possessed distinct redox peaks in the CV spectra, which indicated that the electrocatalytic activity of *P. stutzeri* S116 was associated with the electrode. The bioanode exhibited two distinct reduction peaks (− 0.92 mA at − 0.504 V, − 0.845 mA at − 0.665 V) and the highest oxidative peak current of 0.595 mA at 0.308 V. The biocathode exhibited exhibited three distinct oxidation peaks (− 0.13 mA at − 0.488 V, 0.246 mA at − 0.354 V, 0.105 mA at − 0.24 V) and reduction peaks (− 1.1 mA at − 0.795 V, − 0.743 mA at − 0.62 V, − 0.584 mA at − 0.52 V). Simultaneously, for bare bioanode, no distinct redox reaction was measured. The position of the redox peak reflects the redox potential of components involved in extracellular electron transfer (EET) [54]. In addition, the size of the redox peak represents the electrochemical activity of the bioelectrode using *P. stutzeri* as biocatalyst.

Polarization and power density curves of the MFCs were tested during the third cycle when the Reactors generated voltage at the highest point (shown in Fig. 3 d). The obtained maximum power was 765 mW/m^2^ (Reactor 1) and 656.6 mW/m^2^ (Reactor 2).

The interaction between the electrogenic microbe and the electrodes in MFCs was analyzed by EIS. The Nyquist plot (Supplementary Fig. 1) showed that the biocathode electrode had a smaller semicircle diameter, which represented a lower charge-transfer resistance (Rct) and better catalytic reaction and electron transfer. The Rct values of biocathode and bioanode in the MFCs were approximately 11.8 and 17.0 Ω, respectively. Biocathode can significantly improve the electron transfer rate between biofilms and electrodes, reduce impedance, effectively enrich some electricity-producing microorganisms, and therefore improve the performance of MFCs.

CV data indicated that at least two kinds of extracellular components were secreted by strain S116, which was also supported by gene function analysis (many genes encoding riboflavin and phenazine synthesis were detected in Nr, GO, VFDB and KEGG).

### Genomic features of *P. stutzeri* S116

**Fig. 4:**
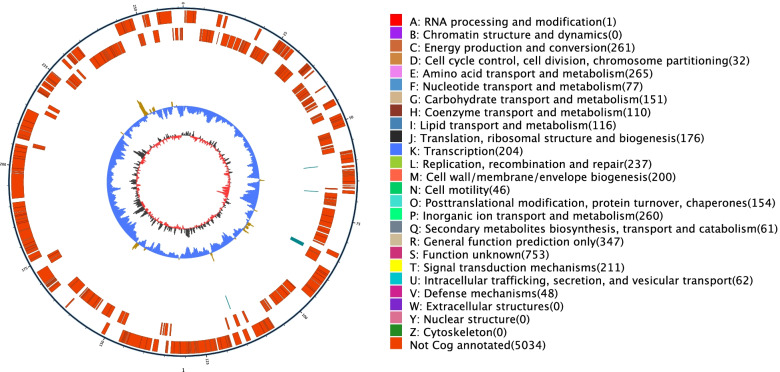
Schematic of the complete genome of *P. stutzeri* S116 isolated from marine activated sludge samples. The first circle (outermost) indicates genomic numbers, with each tick representing 5 kb; genes on forward and reverse chains with different colors based on COG categories are represented at the second and third circles; repetitive sequences (fourth circle); tRNA with bule and rRNA with purple (fifth circle); GC skew (sixth circle). The lightly yellow region indicates that the GC content is higher than the average in the genome; nevertheless, the bule region represents the opposite. The dark gray region represents G content greater than C, and the red region represents C content greater than G

The filtered subreads of the *P. stutzeri* S116 genome were assembled and rectified into a scaffold length of 4,756,665 bp with a GC content of 63.47%. Gene prediction indicated a total gene length of 4,224,096 bp with 4402 CDSs. 3842, 3371, 2493, 4385 and 2805 functional genes of the strain were annotated in eggNOG (COG), GO, KEGG, NR, and SwissProt databases, respectively. In addition, 121, 1343 and 887 genes were annotated in CAZy, TCDB, and VFDB databases, respectively.. Schematic of the complete genome of *P. stutzeri* S116 was shown in Fig. 4. The genome sequences are publicly available in the NCBI database (BioProject accession PRJNA743140).

### Gene function analysis

The protein sequences of genes were aligned against Nr database by BLAST, species distribution was exhibited in Supplementary Fig. 2. Three thousand seven hundred forty-four genes are responsible for *Pseudomonas stutzeri* with the highest proportion (85.38%).

**Fig. 5:**
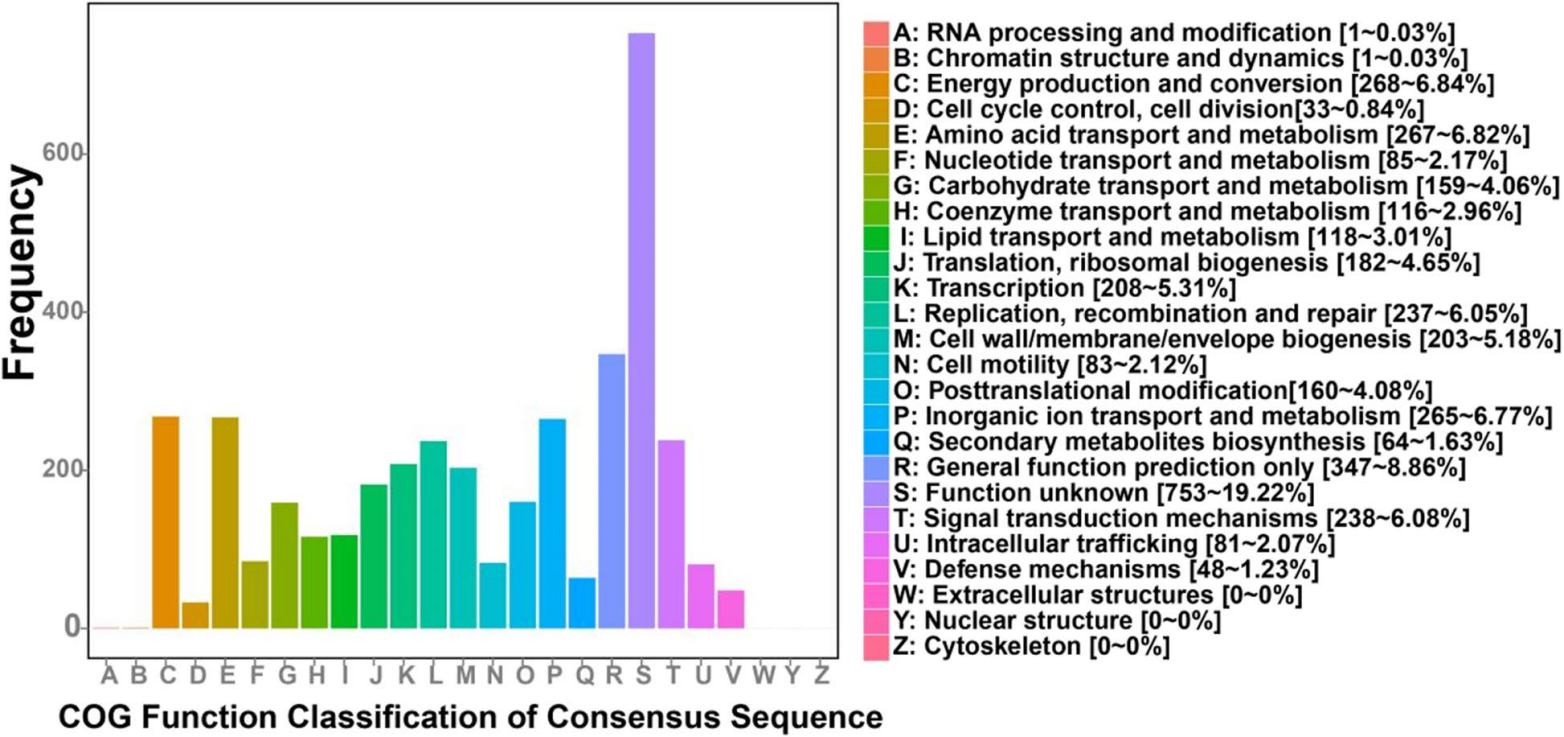
Functional categories of *P. stutzeri* S116 annotated by clusters of orthologous groups of proteins (COGs)

In the COG categories, energy production and conversion (268 genes), amino acid transport and metabolism (267 genes), and inorganic ion transport and metabolism (265 genes) had higher abundances, with proportions of 6.84, 6.82, and 6.77%, respectively (shown in Fig. 5). To detect the potential roles of *P. stutzeri*, specific COGs involved in bioelectricity generation were analyzed. For energy production and conversion, dehydrogenase (COG0508, COG1012, COG1052, COG1063, COG1071, COG1319, NOG00108, NOG02207), cytochrome c (COG3258, COG2010, COG3909, NOG62129, NOG18013) and electron transport complex (COG2878, COG4657, COG4658, COG4659, COG4660) were the three most abundant gene function class, which are all involved in electron transport [20]. Simultaneously, the important components of the respiratory chain, such as complex I (NOG31185, NOG34255), Fe-S protein (COG2975, COG3313), NADH dehydrogenase (COG1252), succinate dehydrogenase (COG0479, COG1053), cytochrome b561 (COG3038), and complex III (COG0723, COG1290), were annotated. Moreover, cytochrome c oxidase (COG2993, COG4736) playing an important component of complex IV had been detected, which could reduce oxygen to water as the terminal electron acceptor in the respiratory chain [55]. With respect to amino acid transport and metabolism function and inorganic ion transport and metabolism, ATP-binding cassette (ABC) transporter (COG0410, COG0411, COG0559, COG0834, COG4160, COG4177, COG4215) was relatively higher abundant. This gene is a virulent gene of *Pseudomonas*, which contribute to resisting severe environment.

Genes of *P. stutzeri* were categorized by GO into three functional nodes to determine the biological relevance of the strain In the three GO categories, biological process was the most abundant, and molecular function was the least abundant (Supplementary Fig. 3).

In the biological process category, genes involved in metabolic processes (1737 genes) made up the highest proportion (51.5%) of the total genes (3372 genes), cellular process (1497 genes; 44.4%), single-organism process (1312 genes; 38.9%), and localization (536 genes; 15.9%). In the molecular function category, most genes of 1918 were involved in catalytic activity, with a proportion of 56.9%, and in binding, with a proportion of 43.5% (1467 genes). In the cellular component category, 1140 genes involved in membrane had the highest proportion of 33.8%, membrane part (1040) 30.8%, cell (1030) 30.5%, and cell part (1007) 29.9%.

For *P. stutzeri* S116, the five most abundant genes were annotated in the VFDB (Supplementary Fig. 4), including type IV pili (61 genes), capsule (49 genes), flagella (44 genes), pyoverdine (38 genes) and polar flagella (37 genes). Sixty-one genes encoding type IV pilus biogenesis proteins were annotated (6.88% of the total VFDB annotations), and the conductive pilus of electrogenic microorganisms is one of the important mechanisms of EET, such as *Geobacter sulfurreducens*, which transports electrons through its pilus belonging to type IV pili [56, 57]. Pyoverdine contributes to the survival of microbes in nutrient-deficient soil [58].

### Critical metabolic pathways

Genes were annotated against the KEGG databases to investigate the critical metabolic pathways involved in anodic and cathodic catalysis in MFCs. For *P. stutzeri* S116, energy metabolism and a two-component system are the two essential functions in KEGG annotations (shown in Supplementary Fig. 5).

The respiratory chain on the membrane of *P. stutzeri* S116 is an important pathway for electron transport and energy production. Oxidative phosphorylation (ko00190, 36 genes) indicates that strain S116 possesses an integrated electron transport chain, and critical enzymes were detected, including succinate dehydrogenase (EC:1.3.5.1), ubiquinol-cytochrome c reductase (EC:1.10.2.2) and cbb3-type cytochrome c oxidase (EC:7.1.1.9, cytochrome aa3). In complex II of the respiratory chain, *sdhC* (K00241), *sdhD* (K00242), *sdhA* (K00239) and *sdhB* (K00240) encode cytochrome b, membrane anchor subunit, iron-sulfur subunit and flavoprotein subunit, respectively, where succinate is dehydrogenized into fumarate. Complex III primarily contains ubiquinol-cytochrome c reductase iron-sulfur subunit (EC:7.1.1.8), ubiquinol-cytochrome c reductase cytochrome b subunit (K00412) and ubiquinol-cytochrome c reductase cytochrome c1 subunit (K00413). The electrons are transported from complex III to cytochrome c oxidase (complex IV) through cytochrome c, where oxygen is reduced into H_2_O and energy is generated. Nevertheless, annotated type 2 NADH dehydrogenase (K03885, EC:1.6.99.3) is involved in regulation rather than respiration [59]. Therefore, electron transport in *P. stutzeri* S116 forms a succinate pathway with high probability (Supplementary Fig. 6).

Generally, there are two oxidation pathways from thiosulfate to SO_4_^2−^ or S_4_O_6_^2−^ in SOB. (1) S_2_O_3_^2−^ is oxidized to SO_4_^2−^ by the Sox multienzyme complex [60]. (2) Thiosulfate dehydrogenase (EC:1.8.2.2, tsdA) catalyzes S_2_O_3_^2−^ to S_4_O_6_^2−^ [61]. In addition, the pathway of sulfur metabolism (ko00920, 34 genes) indicates that thiosulfate is catalyzed by thiosulfate sulfurtransferase (EC:2.8.1.1) into sulfite. Moreover, the *sqr* gene encoding sulfide:quinone oxidoreductase (EC:1.8.5.4) was detected, which can oxidate H_2_S into S_0_.

Riboflavin can freely shuttle cell membranes and capture electrons from the respiratory chain, which plays an important role in EET. Riboflavin metabolism (ko00740 8 genes) for *P. stutzeri* indicates that ribulose 5-phosphate is metabolized into riboflavin. In addtion, riboflavin, as a redox active compound, is secreted by many bacteria [62]. COG0307 and COG0196 encoding riboflavin synthase and riboflavin kinase are annotated in COG, which are essential enzymes related to the biosynthesis of riboflavin.

**Fig. 6:**
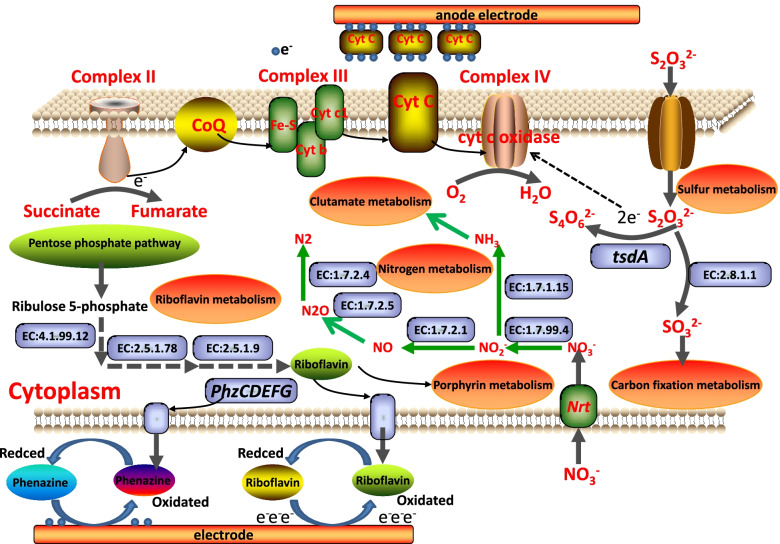
Metabolic pathways for *P. stutzeri* S116 involved the electron respiratory chain, nitrate reduction pathway, thiosulfate oxidation pathway, riboflavin metabolism and the predicted EET pathway between the electronic mediators and the electrodes

Pili are generally detected in gram-negative bacteria and are closely related to bacterial activity, biofilm formation, surface adhesion, DNA acquisition and signal transduction [63]. Genes encoding type IV pilus-assembly proteins, such as *pilB*, *pilC*, *pilE*, *pilW*, *pilZ*, *pilV*, *pilO*, *pilM*, *pilN*, *pilQ*, *pilY*, *pilV* and *pilP*, were detected in the COG and KEGG databases. The many genes encoding pilin imply that the type IV pilus of *P. stutzeri*, as an anodic electricity-producing bacterium, probably plays an important role in EET. Two-component system (ko02020, 153 genes) proteins from *P. stutzeri* involved in chemotaxis primarily include twitching motility proteins encoded by genes such as *pilG*, *pilH*, *pilI*, *pilJ*, and *pilK*. Moreover, the redox signal is transmitted by the annotated critical sensor histidine kinase (EC:2.7.13.3, K15011) into an electron transfer system and aerobic respiration. Simultaneously, a two-component system indicated that nitrate and nitrite were phosphorylated, transported to nitrate reductase, and finally entered the nitrogen metabolism pathway (ko00910, 36 genes). The predicted metabolic pathways in *P. stutzeri* were shown in Fig. 6.

## Discussion

*Pseudomonas* is a typically electrogenic microorganism used in MFCs as anodic biocatalyst. It was tested for its prominently electrochemical activity in the anolyte zone of the MFCs [64]. However, for biocathode MFCs, few studies have described it comprehensively and completely. in this study, the strain S116 exhibited excellent performance as a biocathode with prominent redox activity. The low Rct (11.8 Ω) values of biocathode would be favorable for large-scale application and it indicates that S116 is a highly efficient catalyst between the biofilm and the cathode electrode. Moreover, the cost of biocathode MFCs are distinctly lower than abiotic MFCs (such as transition metal elements, Pt-coated metals, and ferricyanide). Simultaneously, biocathodes can improve MFCs sustainability because consumption of electron mediator will be solved [36]. In a word, *P. stutzeri* S116 is a promising electrogenic microorganism owning bifunctional catalysis used in MFC which has never been reported.

Many genes encoding cytochrome c contribute to generating electricity according to COG function analysis, which can form a complex extracellular electron transport network and realize the transmembrane transport of electrons [18, 65, 66]. Simultaneously, type IV pilus as “Nanowires” were detected. However, the truncated pilus protein (*pilA* encoding) is not founded in databases, which is closely related to the pilus with high electrical conductivity [67]. Redox mediators such as riboflavin can intercept electrons from the respiratory chain, and transfer them outside the cell membrane [68]. Riboflavin metabolism pathway in *P. stutzeri* indicates 8 critical genes involved in riboflavin synthesis. Riboflavin synthase (EC:2.5.1.9) in the reaction process is detected, which catalyzes the last step of riboflavin biosynthesis in microorganisms. Furthermore, riboflavin is synthesized into dimethyl-benzimidazole (entering porphyrin and chlorophyll metabolism) or FAD. In the VFDB, six genes (*phzF1*, *phzC1*, *phzG1*, *phzH*, *phzE*1 and *phzD*1) are responsible for phenazine biosynthesis. Phenazine secreted by *P. aeruginosa* is a heterocyclic compound containing nitrogen that plays an important role in EET as a physiological electron transfer mediator of electricigens [69]. In this study, the CV curve showed that definite reduction and oxidation peaks were detected in the range of − 0.7 V ~ 0 V (vs. Ag/AgCl), which approaches the redox potential of phenazine and riboflavin [70]. Due to the lack of an intact *Sox* complex in *P. stutzeri*, thiosulfate oxidation probably performs the pathway where S_2_O_3_^2−^ is catalyzed to S_4_O_6_^2−^ by tsdA. However, the protein *tsdB* is considered to the electronic acceptor in *Pseudomonas stutzeri* A1501 [71], which was not detected in *P. stutzeri*. *tsdA* probably acts as an electron acceptor to oxidize S_2_O_3_^2−^ in some SOB.

The nitrogen metabolism pathway implies that *P. stutzeri* can be used in treating wastewater containing nitrite. Nitrate can be reduced into nitrogen through denitrifying bacteria. The nitrogen metabolism pathway indicates that functional denitrification genes such as *nirS* (playing an important role in nitrite reduction), *norB*, *nosZ*, and *narG* [72] are detected in *P. stutzeri* with denitrification. In addition, nitrate is metabolized into ammonia through dissimilatory nitrate reduction, and the annotated gene *nirBD* encodes nitrite reductase (EC:1.7.1.15) and can contribute to nitrite reduction [73].

## Conclusions

The present study provided a promising bifunctional biocatalyst used in MFCs compared with abiotic MFCs. Complete genome sequence of *Pseudomonas stutzeri* S116 and CV data represent redox mediators secreted by *P. stutzeri* S116 were probably responsible for performance of MFCs. The critical genes and metabolic pathways involved in thiosulfate oxide and nitrate reduction were detected, which indicated that the strain can treat wastewater containing sulfide and nitrite more efficiently.

## Supplementary Information

**Additional file 1: Supplementary Fig. 1.** EIS Nyquist plots of the anode and cathode electrode in MFCs. **Supplementary Fig. 2.** The protein sequences of genes were aligned against Nr database by BLAST. **Supplementary Fig. 3.** GO Classification for *P. stutzeri* S116 isolated from marine activated sludge. The chart shows the enriched genes with secondary-level functions in all genes against GO. **Supplementary Fig. 4.** The nine most abundant virulence factors annotated in *P. stutzeri* S116. Phenazine biosynthesis factor indicates genes encoding phenazine generation, which is an important electronic mediator investigated in the genus *Pseudomonas*. HitABC represents ABC transporter and ATP-binding protein, AcfB represents accessory colonization factor AcfB. **Supplementary Fig. 5.** Genes were annotated against the KEGG databases. **Supplementary Fig. 6.** Electron transport in *P. stutzeri* S116 forms a succinate pathway with high probability.

## Data Availability

The datasets generated and/or analysed during the current study are available in the Treebase repository and NCBI database. Phylogeny data (including alignments). http://purl.org/phylo/treebase/phylows/study/TB2:S28911?x-access-code=52f486836b34e93c0c34658911e7e960&format=html Gene data. 16S sequence data of *P. stutzeri* S116. Genbank accession number MZ220459. https://www.ncbi.nlm.nih.gov/nuccore/MZ220459.1/ Genomic data of *P. stutzeri* S116. BioProject Accession PRJNA743140. https://www.ncbi.nlm.nih.gov/bioproject/743140/
