# Supplementary material for: Complete genome sequence of Pseudomonas stutzeri S116 owning bifunctional catalysis provides insights into affecting performance of microbial fuel cells
Source: BMC Microbiol. 2022 May 19;22:137. doi: 10.1186/s12866-022-02552-8 (PMC9118636; doi:10.1186/s12866-022-02552-8)
Supplement: Supplementary file 1 — Additional file 1: Supplementary Fig. 1. EIS Nyquist plots of the anode and cathode electrode in MFCs. Supplementary Fig. 2. The protein sequences of genes were aligned against Nr database by BLAST. Supplementary Fig. 3. GO Classification for P. stutzeri S116 isolated from marine activated sludge. The chart shows the enriched genes with secondary-level functions in all genes against GO. Supplementary Fig. 4. The nine most abundant virulence factors annotated in P. stutzeri S116. Phenazine biosynthesis factor indicates genes encoding phenazine generation, which is an important electronic mediator investigated in the genus Pseudomonas. HitABC represents ABC transporter and ATP-binding protein, AcfB represents accessory colonization factor AcfB. Supplementary Fig. 5. Genes were annotated against the KEGG databases. Supplementary Fig. 6. Electron transport in P. stutzeri S116 forms a succinate pathway with high probability. [file 12866_2022_2552_MOESM1_ESM.docx]

Supplementary Material for:

BMC Microbiology

**Complete genome sequence of Pseudomonas stutzeri S116 owning bifunctional catalysis provides insights into affecting performance of microbial fuel cells**

**Peng Li^1*^ · Wenfeng Yuan^1*^ · Yitie Huang^1*^ · Caiyu Zhang^1^ · Chide Ni^1^ · Qi Lin^2^ · Zhihuang Zhu^2^ · Jianxin Wang#^1^**

^1^School of Ocean Science and Technology, Zhejiang Ocean University, Zhoushan, Zhejiang 316022, China

^2^Fisheries Research Institute of Fujian Province, Xiamen 361013, China

*These authors contributed equally

**^#^** Corresponding author

corresponding author:

No.1, Haida South Road, Lincheng Changzhi Island, Zhoushan, Zhejiang, P.R.China

[wangjianxin3818081@163.com](mailto:Wangjianxin3818081@163.com), [43677860@qq.com](mailto:lpqp3818081@163.com) +86 580 2551332

**Supplementary Figure 1:** EIS Nyquist plots of the anode and cathode electrode in MFCs.


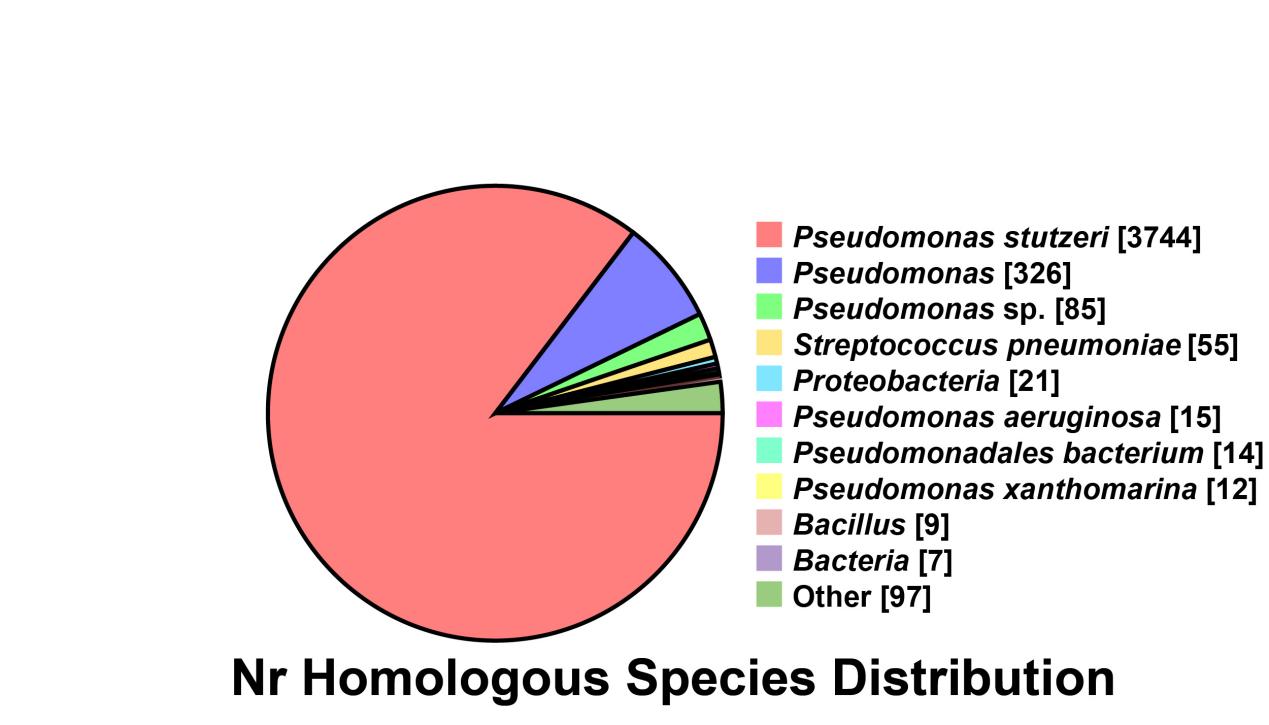


**Supplementary Figure 2:** The protein sequences of genes were aligned against Nr database by BLAST


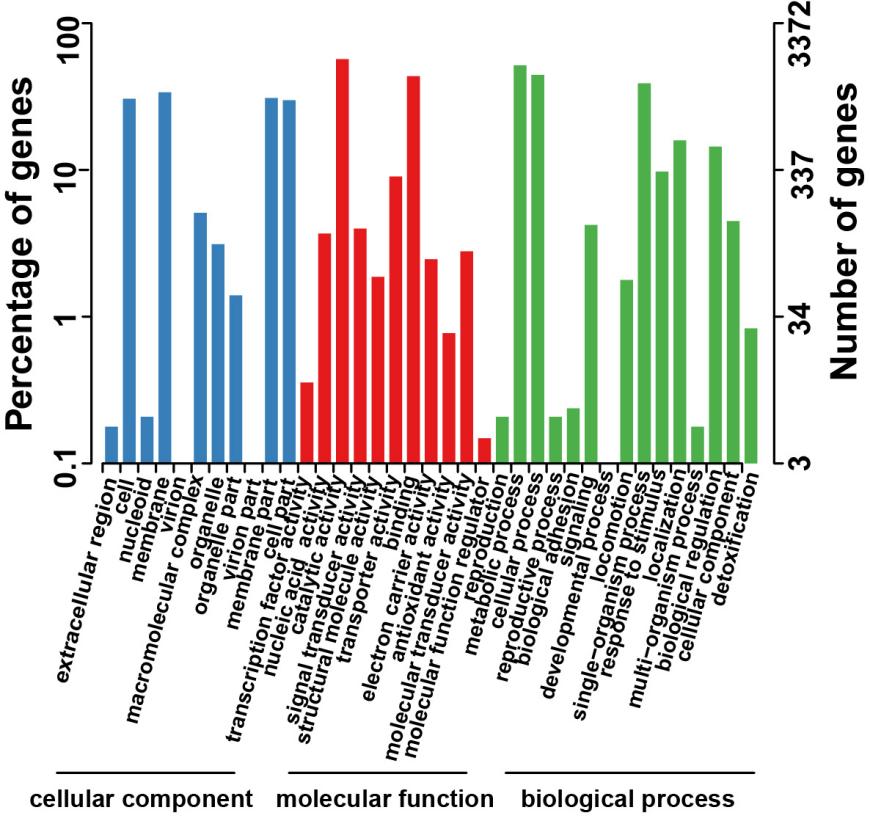


**Supplementary Figure 3:** GO Classification for *P. stutzeri* S116 isolated from marine activated sludge. The chart shows the enriched genes with secondary-level functions in all genes against GO.

**Supplementary Figure 4:** The nine most abundant virulence factors annotated in *P. stutzeri* S116. Phenazine biosynthesis factor indicates genes encoding phenazine generation, which is an important electronic mediator investigated in the genus *Pseudomonas*. HitABC represents ABC transporter and ATP-binding protein, AcfB represents accessory colonization factor AcfB.


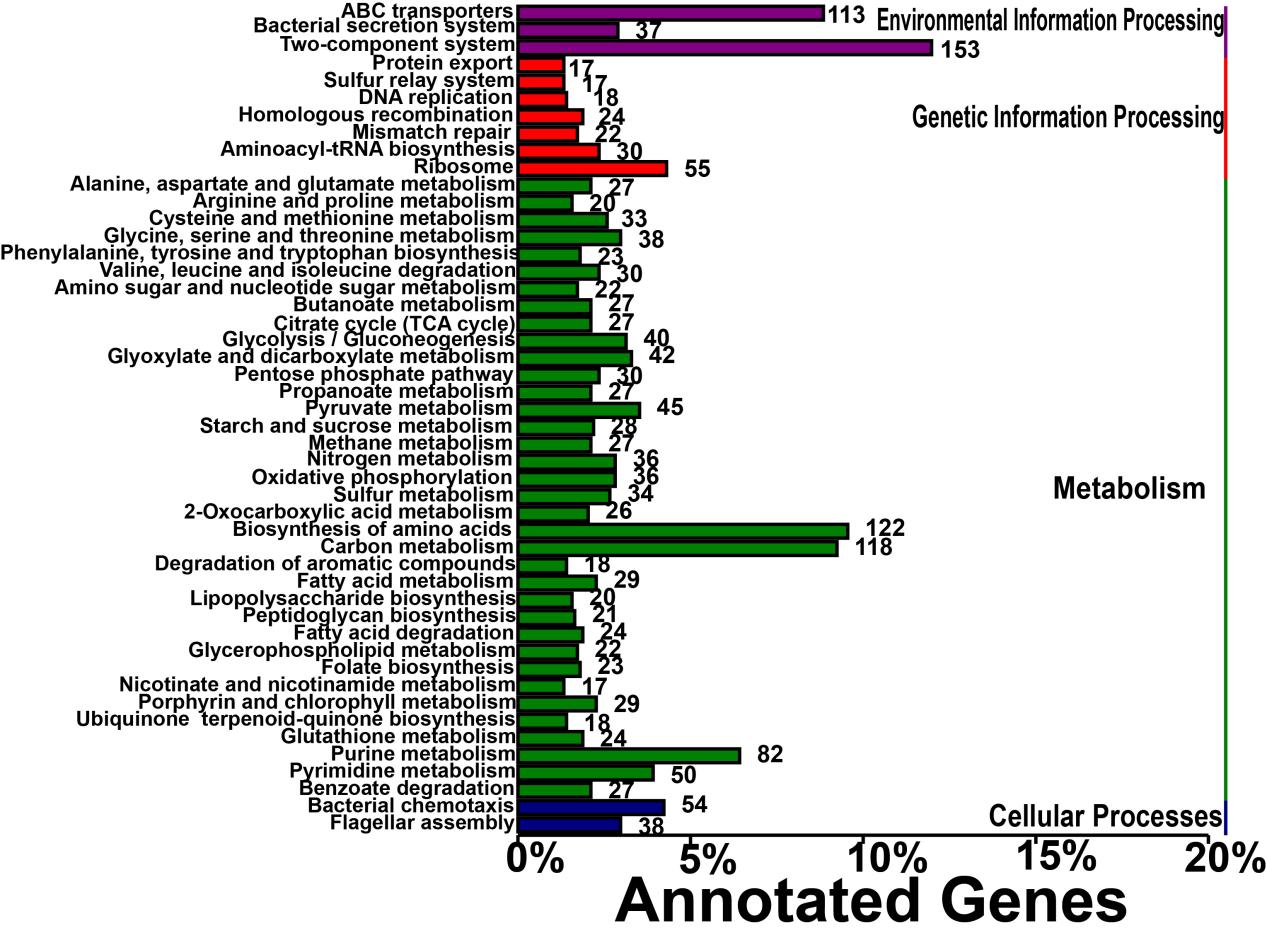


**Supplementary Figure 5:** Genes were annotated against the KEGG databases.


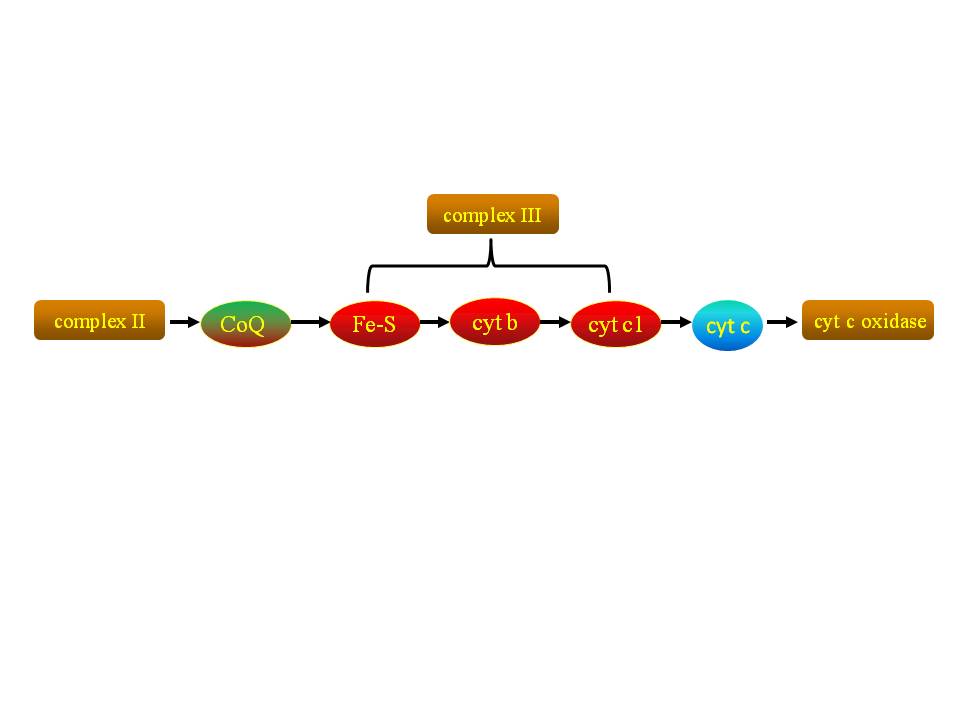


**Supplementary Figure 6:** Electron transport in *P. stutzeri* S116 forms a succinate pathway with high probability.
